# Effectiveness of benznidazole in the treatment of Chagas disease in children and adults: a long-term retrospective cohort study

**DOI:** 10.1128/aac.00203-26

**Published:** 2026-09-11

**Authors:** A. Rabinovich, C. Cruz, G. Moscatelli, S. Moroni, N. González, J. C. Ramirez, F. Garcia-Bournissen, G. Ballering, H. Freilij, J. Altcheh

**Affiliations:** 1 Servicio de Parasitología and Instituto Multidisciplinario de Investigaciones en Patologías Pediátricas (IMIPP), CONICET-GCBA, Hospital de Niños Ricardo Gutierrez, Buenos Aires, Argentina; 2 Division of Clinical Pharmacology and Toxicology, Department of Pediatrics, Sickkids, The Hospital for Sick Children, University of Toronto, Toronto, Ontario, Canada; 3 Mahidol Oxford Research Unit (MORU), Centre for Tropical Medicine and Global Health, Nuffield Department of Medicine, University of Oxford 6396, Oxford, United Kingdom; 4 Division of Paediatric Clinical Pharmacology, Department of Paediatrics, Schulich School of Medicine and Dentistry, Western University 6221, London, Ontario, Canada; The Children's Hospital of Philadelphia, Philadelphia, Pennsylvania, USA

**Keywords:** *Trypanosoma cruzi*, treatment, benznidazole, children, adults, Chagas disease

## Abstract

Chagas disease, caused by *Trypanosoma cruzi*, is endemic to the Americas but has spread worldwide due to migration. Benznidazole is effective, but long-term follow-up efficacy studies are scarce and include only small numbers of patients. We analyzed data from a retrospective cohort of Chagas disease patients treated with benznidazole at a large tertiary center in Argentina (1980–2019). Treatment response was assessed through clinical, parasitological, and serological follow-up. Parasitemia was evaluated by direct visualization or PCR. Among 567 treated patients, 464 met selection criteria, including 411 children (median age: 46 months, IQR: 9.5–124 months) and 53 adults (median age: 26 years, IQR: 20–34 years). At diagnosis, 11/411 (2.68%) children and 1/53 (1.9%) adults were symptomatic; all improved after treatment. Baseline parasitemia was detected in 289/445 (64.94%) of patients, and 279/289 (96.54%) had parasitological follow-up for at least 3 years. Median benznidazole dose was 6.6 mg/kg/day in children and 5.6 mg/kg/day in adults, with treatment durations of 8 weeks and 31 days, respectively. All patients cleared parasitemia at treatment completion. Median serological follow-up was 4.8 years for children and 2.8 years for adults. At 1-year follow-up, 38.8% of patients showed >20% reduction in *T. cruzi* antibody titers, and 29.8% achieved seronegativity, with younger patients converting faster (*P* < 0.01). In this Argentinian cohort, Benznidazole achieved sustained parasitemia clearance and declining antibody titers, supporting early treatment initiation.

## INTRODUCTION

Chagas disease is caused by the kinetoplastid protozoa *Trypanosoma cruzi*. Chagas disease is a condition characterized by chronicity and social stigma, and is closely linked with poverty. It is endemic in 21 countries of Latin America and has spread with the Latin American diaspora (1). Over six million people are infected. Chagas disease remains neglected despite its severity (2).

Chagas disease is spread by various species of blood-feeding reduviid insects, commonly called “kissing bugs.” In the form transmitted by these vectors, nearly 90% of individuals exhibit no symptoms during the acute stage. However, many decades later, approximately 20% of patients develop clinical manifestations characterized by severe involvement of the cardiovascular, gastrointestinal, and sometimes nervous systems (3). *T. cruzi* transmission can also occur through organ donation, blood transfusion, laboratory accidents, orally via consumption of contaminated food or drinks, and vertical transmission, which is currently the leading cause of transmission in areas with vector control (4, 5).

The World Health Organization (WHO), through Pan American Health Organization (PAHO) guidelines (6, 7), has stated that all patients with acute Chagas disease, and most patients with chronic disease, should receive antiparasitic treatment. Unfortunately, the current estimation is that less than 1% (8) of infected patients are receiving treatment. There are many obstacles: low awareness of Chagas disease by healthcare workers and Chagas disease treatment options, a limited understanding of the disease biology, drug shortages or unavailability, the requirement for extended treatment periods, a poorly characterized PK-PD profile, and the reportedly high incidence of severe adverse events. New drugs are being developed to treat Chagas disease (9, 10) but, even if they survive, they will not be available in the near future. Benznidazole and nifurtimox, both over 50 years old, are the only two available medications to treat this condition, each with a distinct adverse event profile but with no reported difference in effectiveness.

Chagas disease should also be considered a pediatric disease, as many infections occur during childhood, with congenital transmission being the primary route of infection, particularly in urban areas without vector transmission. Despite evidence of benznidazole’s efficacy in children, until 2011, there was no pediatric formulation. The only available dosage form was a 100-mg tablet, suitable for adults (11). Benznidazole was added to the WHO Essential Medicines List in 1987, and the pediatric formulation (12.5 mg tablet) was included in the 2013 update (12, 13).

Assessing therapeutic response in Chagas disease presents challenges and creates controversies, primarily because of the difficulty in establishing cure. This results from the prolonged persistence of *T. cruzi*-specific antibodies, the limited sensitivity of parasitological tests in chronic disease (14), and ongoing debates about parasite clearance in the indeterminate phase that prevents later end-organ damage. Achieving seronegativity in conventional *T. cruzi* serological tests requires a prolonged follow-up of treated patients. Conducting long-term follow-up efficacy studies requires infrastructure and resources that are often scarce in settings where Chagas disease is prevalent. There are few reported long-term cohorts of patients treated for Chagas disease (15–21), some of the largest originating from our group.

Here, we describe and compare the efficacy of benznidazole across age groups based on data from a large cohort of Chagas disease–treated patients in Argentina, including infants, children, and adults who received treatment from January 1987 to July 2019. To our knowledge, this is one of the largest studies of Chagas disease describing an extended longitudinal observation period with both parasitological and serological outcomes.

## RESULTS

### Population and treatment received

**Fig 1:**
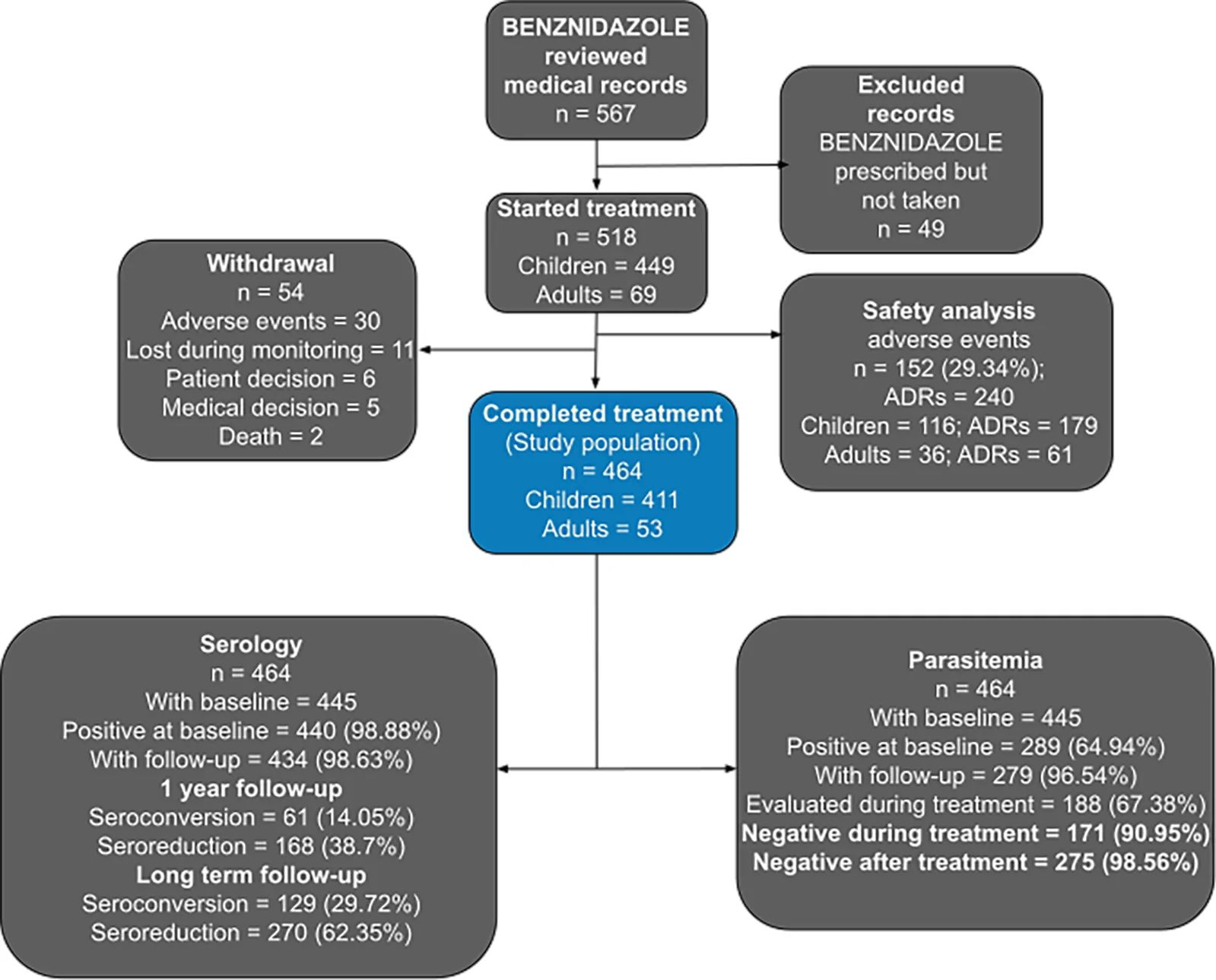
Flow diagram.

After reviewing the medical records of Chagas disease patients treated at our institution, we identified 567 prescribed benznidazole patients. After applying the selection criteria, 464 patients (411 children and 53 adults) were included (Fig. 1, blue box).

The distribution of male and female subjects was balanced in children but not in adults, where, as explained, 75.5% were female. The parasite transmission was congenital in 268 (57.75%), undetermined in 167 (36%), vectorial in 27 (5.8%), and by blood transfusion in 2 (0.4%) cases (Table 1).

**TABLE 1:** Demographic data

	(0–7 mos) Patients (%) *n* = 66 (14.2)	(8 mos–1yr) Patients (%) *n* = 99 (21.3)	(2–6 yrs) Patients (%) *n* = 83 (17.9)	(7–11 yrs) Patients (%) *n* = 85 (18.3)	(12–17 yrs) Patients (%) *n* = 78 (16.8)	(Over 18 yrs) Patients (%) *n* = 53 (11.4)	Total patients *n* = 464
Gender							
F	30 (45.5)	56 (56.6)	39 (47)	40 (47.1)	38 (47.7)	40 (75.5)	243 (52.4)
M	36 (54.5)	43 (43.4)	44 (53)	45 (52.9)	40 (51.3)	13 (24.5)	221 (47.6)
Age (months)							
Median [Q1, Q3]	1.00 [1.00, 3.00]	11.0 [9.0, 13.5]	46 [29.5, 60]	109 [96.0, 131]	168 [155, 180]	312 [240, 408]	65.5 [11, 144]
Mean (SD)	2.48 (2.23)	11.63 (3.58)	46.12 (17.09)	111.99 (18.47)	167.22 (17.86)	335.66 (116.7)	98.05 (111.30)
Min-Max	1.00–7.00	8.00–23.0	24.0–82.0	84.0–143	144–213	216–708	1.00–708
Route of infection							
Vectorial	4 (6)	1 (1)	1 (1.2)	2 (2.35)	10 (12.82)	9 (16.98)	27 (5.82)
Congenital	62 (93.94)	88 (88.9)	53 (63.86)	36 (42.35)	21 (26.92)	8 (15.1)	268 (57.76)
Blood transfusion	0	0	0	1 (1.17)	0	1 (1.88)	2 (0.43)
Undetermined	0	10 (10.1)	29 (34.94)	46 (54.12)	47 (60.26)	35 (66.04)	167 (35.99)

Median benznidazole dose for children was 6.6 mg/kg per day (IQR: 5.7–7.3 mg/kg) in two doses with a median treatment length of 60 days (IQR: 60–60 days). In adults, the median benznidazole dose was 5.6 mg/kg (IQR: 5.2–6.1 mg/kg) in two doses with a median treatment length of 31 days (IQR: 30–60 days). Treatment was well tolerated, and good treatment compliance was observed. Details of adherence and observed ADRs were described in a previous publication (22).

Median serological follow-up time for children was 4.8 years or 58.2 months (IQR: 22.4–111.5 months), with retention during long-term follow-up of 91% at 5 months after treatment, 82% at 12 months, 74% at 24 months, 66% at 36 months, and 49% at 60 months.

Adults had a shorter follow-up time with a median of 2.8 years or 33.8 months (IQR: 10.3–67.9 months), with retention during long-term follow-up of 85% at 5 months after treatment, 72% at 12 months, 55% at 24 months, 44% at 36 months, and 30% at 60 months. The median follow-up time for parasitemia monitoring using PCR in pediatric patients was 29.5 months (IQR: 2–55.3 months), while for adults, it was 21.8 months (IQR: 6–36 months).

### Clinical

Most patients were asymptomatic at diagnosis. Only 11/411 children (2.68%) and 1/53 (1.9%) adults were symptomatic. Nearly all symptomatic cases (9 cases) were in children between 0 and 8 months.

The only symptomatic adult had contracted Chagas disease through a vectorial route, was co-infected with HIV, and was diagnosed during hospitalization. This case has been reported previously (23).

Overall, 2/17 (11.8%) of children infected via the vectorial route were symptomatic, compared to 9/261 (3.44%) of those infected via the congenital route. All patients with undetermined routes of infection, 127 children and 33 adults, were asymptomatic.

Among patients infected via the congenital route, symptoms included enlarged liver (5/9), jaundice (3/9), and enlarged spleen (1/9). One patient had low birth weight (1/9). Patients infected via the vectorial route, including the single adult patient, presented chagoma (1/3), cerebral chagoma (1/3), splenomegaly (1/3), and meningoencephalitis (1/3) (Table 2). All symptomatic patients had clinical improvement during treatment.

**TABLE 2:** Clinical findings in symptomatic patients (vector-borne and congenital)^*a*^

Route of infection/clinical finding	No. of patients (%)
Vector borne	*n* = 26
Clinical examination	
Asymptomatic	23 (88.46)
Symptomatic	3 (11.54)
Symptoms	
Chagoma	1 (25)
Meningoencephalitis	1 (25)
Cerebral chagoma in CT scan	1 (25)
Splenomegaly	1 (25)
Congenital	*n* = 269
Clinical examination	
Asymptomatic	260 (96.65)
Symptomatic	9 (3.35)
Symptoms	
Jaundice	3 (27.27)
Neonatal hepatitis	3 (27.27)
Hepatomegaly	3 (27.27)
Splenomegaly	1 (9.09)
Low weight	1 (9.09)
Undetermined	*n* = 160
Clinical examination	
Asymptomatic	160 (100)

^*a*^ All patients infected by undetermined routes were asymptomatic. Note that a patient may have more than one symptom.

### Cardiological assessment

An electrocardiogram (ECG) was conducted at baseline in 90/465 patients, and 66 of these patients were evaluated every year after treatment follow-up. At baseline, 18/219 displayed alterations in the ECG, but only 11/18 were considered clinically significant. A total of 7/18 patients, mainly infected by the vectorial route, showed clinical and ECG alterations related to acute myocarditis. These patients improved clinically during treatment, and the ECG became normal during follow-up. Four of these 18 patients showed a left bundle branch block presumably related to Chagas disease, and the remaining 7/18 patients showed alterations not related to Chagas disease (four incomplete right bundle branch block [IRBBB], 1 Long QT, 1 Sinoatrial block, and one isolated ventricular extrasystole). During follow-up, two patients with normal ECG at baseline showed ECG alterations (one presented an IRBBB and the other isolated ventricular extrasystoles with an adequate chronotropic range), which were not considered clinically significant.

### Parasitology

Parasitemia was positive in 64.9% (289/445) of patients at baseline in at least one of the following tests: microhematocrit (MH), PCR, xenodiagnosis (XD), mice inoculation, hemoculture (Fig. 1), and 96.5% (279/289) of patients had parasitological follow-up over time. Overall, 98.6% (275/279) cleared parasitemia at the end of treatment. Of the four patients with positive parasitemia at the end of treatment, two showed seroconversion, and one a decrease in *T. cruzi* antibody titers during follow-up (seroreduction). The remaining patient was lost to follow-up. Once negative, parasitemia remained negative during long-term follow-up in 98.9% (272/275) of patients. The remaining three patients oscillated between negative and positive parasitemia during follow-up and were eventually re-treated with nifurtimox. Parasitemia was monitored by real-time PCR during treatment in 67.4% (188/279) of patients, with 91.0% (171/188) achieving clearance before completing therapy. Nineteen patients had been previously treated in other centers. These patients could not provide an exact date for the beginning of the treatment, so they were excluded from serological and parasitology analysis, although their clinical data were still analyzed.

### Serology

At baseline, serology was positive in 98.9% (440/445) of evaluated patients. The five remaining patients were younger than 8 months, showing negative serology but positive parasitemia, confirming congenital infection. A 98.63% (434/440) of patients with a positive baseline serology had serological follow-up over time.

### Seroconversion

**Fig 2:**
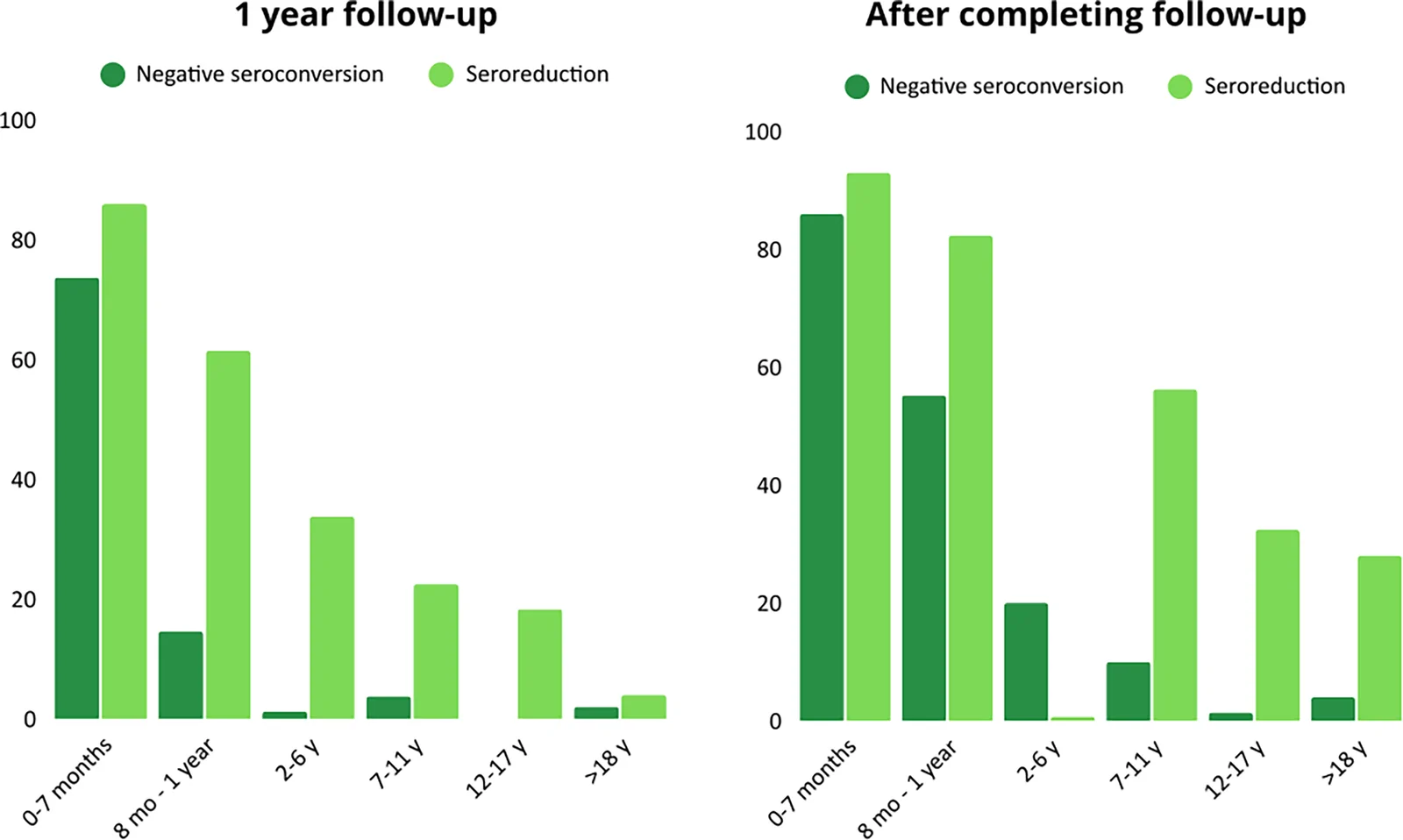
Percentage of patients that showed negative seroconversion (dark green bars) and seroreduction (light green bars) categorized per age at treatment start. This is evaluated in two moments, after 1 year of treatment (left) and after completing follow-up (right).

After 1-year follow-up, 17.37% (61/351) patients showed seroconversion (Fig. 2). The seroconversion rate increased during follow-up. Overall seroconversion was observed in 29.72% (129/434) patients, mainly children, 98.45% (127/129) (Fig. 2 and Table 3). All patients reaching seroconversion also showed negative parasitological results during follow-up, except for two patients who did not have parasitological follow-up after treatment.

**TABLE 3:** Median time to seroconversion and seroreduction stratified by age groups^*a*^

Age group *n* (%)	(0–7 mos) *n* = 57 (13.13)	(8 mos–1 yr) *n* = 96 (22.11)	(2–6 yrs) *n* = 80 (18.43)	(7–11 yrs) *n* = 80 (18.43)	(12–17 yrs) *n* = 71 (16.35)	(Over 18 yrs) *n* = 50 (11.52)	Total *n* = 434 (100)
Seroconversion (%)	49 (85.96)	53 (55.2)	16 (20)	8 (10)	1 (1.4)	2 (4)	129 (29.72)
Median time to seroconversion [95% CI] (mo)	7 (5–8)	46 [34–75]	132 [130–nd]	nd	nd	nd	
Seroreduction (%)	53 (92.98)	79 (82.29)	56 (0.7)	45 (56.25)	23 (32.39)	14 (28)	270 (62.21)
Median time to seroreduction [IQR] (mo)	4 [4–5]	8 [7–11]	22 [14–24]	46 [25–120]	104 [35–nd]	47 [39–nd]	

^*a*^ There was a statistically significant difference in the median times among different age groups (*P* = 0.01; log-rank test). Only patients with positive serology at baseline were included in the analysis. To obtain summary statistics, the “survival” R package was used. It is worth mentioning that median survival time cannot be estimated if less than half of the individuals have an event. In some cases, older age groups may not meet this criterion. In such cases, median survival time is larger than the last reported event and is depicted as an “nd” symbol (no data). To compute median times upper confidence level (UCL95), at least one time point in the survival curve displaying a UCL95 estimation under 0.5 survival probability is required. Older age groups presenting a few events often do not satisfy that criterion. In such cases, the 95% CI depicts an “nd” symbol (no data).

**Fig 3:**
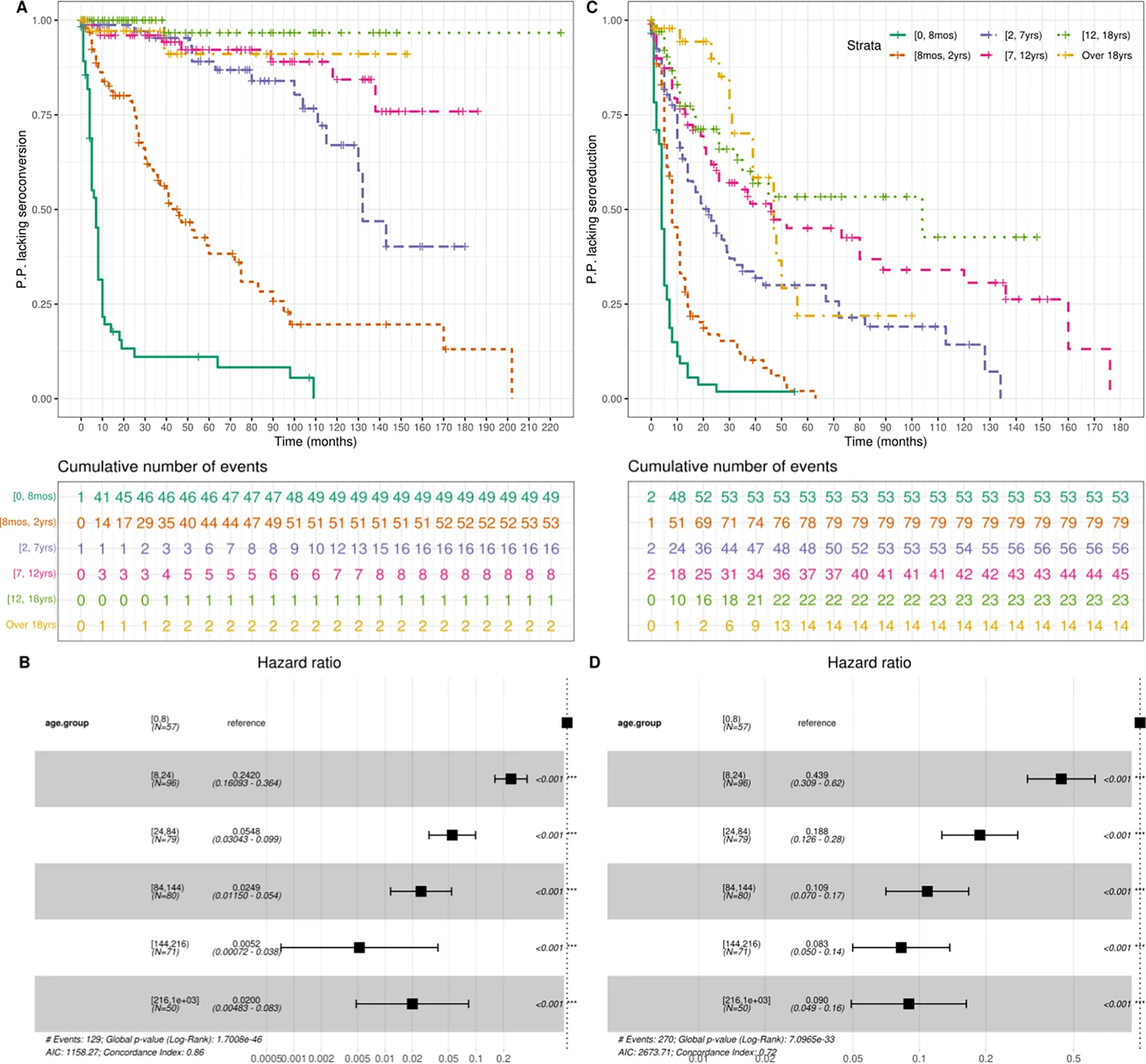
Kaplan–Meier survival curves for seroconversion (**A and B**) and seroreduction (**C and D**) profiles, categorized by age group. The y-axis represents the proportion of patients without seroconversion or seroreduction, while the x-axis represents the time period. The analysis only included patients who had positive serology at baseline, with a total of 277 out of 289 patients (95.8%) included in the analysis. Interestingly, the 0- to 8-month age group had the fastest seroconversion rate. The tables below each curve show the cumulative number of events per age group at each time point, aligned with the x-axis above. Additionally, Cox regression models were used to analyze the seroconversion and seroreduction data, with the infant age group (0–8 months) used as a reference for hazard ratio calculation. The *P* values for comparing hazard ratios among the reference groups are indicated by *, **, and *** for <0.05, <0.01, and <0.001 significance levels, respectively. The analysis was conducted using the “survival” R package version 3.4-0 (30) and the “survminer” R package version 0.4.9.999 (31).

For patients with reactive serology at baseline and follow-up over time (*n* = 434), a Kaplan–Meier survival analysis stratified by age groups was conducted (Fig. 3A). The median time of seroconversion shows differences between age groups (Table 3; *P* = <0.01; log-rank test) and increases with age until 12–17 years. Cox regression models showed that the hazard of seroconversion decreases as the age of the groups increases until 12–17 years (Fig. 3B).

### Seroreduction

At 1-year follow-up (Table 4), overall, 38.7% (168/434) of patients showed seroreduction from baseline antibody titers, mostly children (166/168).

**TABLE 4:** Analysis of seroconversion and seroreduction at 1-year follow-up^*a*^

Age group *n* (%)	(0–7 mos) *n* = 57 (13.13)	(8 mos–1 yr) *n* = 96 (22.11)	(2–6 yrs) *n* = 80 (18.43)	(7–11 yrs) *n* = 80 (18.43)	(12–17 yrs) *n* = 71 (16.35)	(Over 18 yrs) *n* = 50 (11.52)	Total 434 (100)
Seroconversion (%)	42 (73.68)	14 (14.58)	1 (1.25)	3 (3.75)	0	1 (2)	61 (14.05)
Seroreduction (%)	49 (85.96)	59 (61.45)	27 (33.75)	18 (22.5)	13 (18.31)	2 (4)	168 (38.7)

^*a*^ Patients with positive serology at baseline studied at 1 year (at least) of diagnosis.

During long-term follow-up (Table 3), a total of 62.21% (270/434) of patients showed seroreduction. Seroreduction was observed in 256/384 (66.66%) children and 14/50 (28%) adults. The Kaplan–Meier survival analysis stratified by age groups (Fig. 3B) also showed that median seroreduction times were different between the groups, with increasing age being a factor (Table 3, *P* < 0.01; log-rank test). Cox regression models indicated that the hazard of seroreduction decreases as the age of the groups increases (Fig. 3D). In long-term follow-up, antibody reduction increases over time as a marker of treatment response.

### Stratified serological analysis by parasitological baseline profile

**Fig 4:**
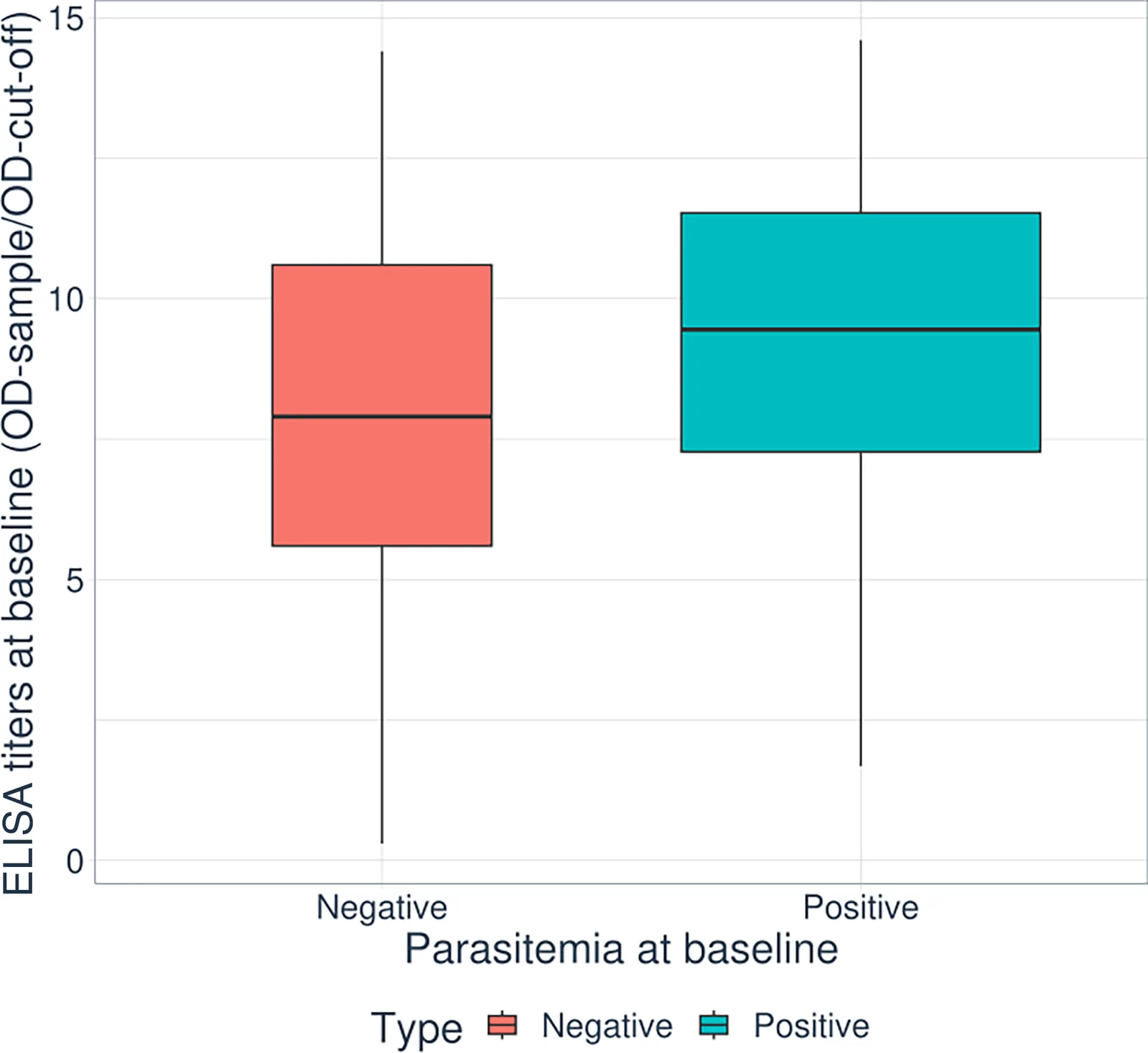
Stratified serological analysis by baseline parasitemia.

A stratified serological analysis according to baseline parasitological status was conducted to assess whether patients with positive parasitemia at baseline had different ELISA titer values compared with those with negative parasitemia at baseline (Fig. 4). Patients younger than 8 months were excluded from this analysis, and comparisons were performed using the Wilcoxon test. Patients with negative parasitemia at baseline had a median ELISA titer of *R* = 7.90 (IQR, 5.60–10.60), whereas those with positive parasitemia had a higher median ELISA titer of *R* = 9.45 (IQR, 7.27–11.52). This difference was statistically significant (*P* < 0.01).

## DISCUSSION

Chagas disease is one of the most neglected parasitic diseases (2). Most infections occur during childhood, and congenital infections are the main route of transmission in urban areas without vector transmission. However, despite existing evidence of the efficacy of the use of BNZ in children (11, 20, 24), the only registered dosage form of benznidazole for the treatment of Chagas disease before 2013 was a 100 mg tablet unsuitable for children. Benznidazole was added to the list of WHO essential medicines in 1987 and updated in 2013, including a 12.5 mg pediatric formulation (13).

In this study, we systematically assessed the efficacy of benznidazole in a large cohort of Chagas disease patients, including both children and adults treated at our institution, a national reference center for the comprehensive care of newborns and pediatric Chagas disease patients in Buenos Aires, Argentina—an area free of vector transmission. Consequently, most children in the study were congenitally infected, and most adult patients were the mothers of these index pediatric cases.

In concordance with the epidemiology described for Chagas disease, most patients were asymptomatic at diagnosis. A favorable clinical treatment effect was observed in the only 12 symptomatic patients, who recovered fully and without clinical relapse during follow-up.

Controversy persists regarding the efficacy of chemotherapy for Chagas disease in asymptomatic patients (25), stemming from a limited understanding of the disease biology, insufficient evidence demonstrating treatment’s preventive effects on end-organ damage, challenges in defining treatment response, and difficulties in determining parasitological clearance post-treatment. It is generally accepted that acute cases, women of childbearing age, newborns, children, and immunosuppressed subjects benefit particularly from antiparasitic treatment, but the role of treatment in adults with chronic indeterminate disease is less clear. Seroconversion is commonly regarded as the marker of parasitological treatment response, but the decline in antibody titers is slow and erratic. Despite consistently negative parasitological findings, seroconversion of *T. cruzi* antibody titers typically occurs several years after treatment, particularly in patients in the late chronic phase (26). This makes treatment assessments difficult.

Declining antibody titers may reflect parasitological cure (27, 28). In our cohort, the markedly lower seroconversion rates observed in adults than in children, particularly infants, are consistent with the known relationship between time to seroconversion and duration of infection (29). Younger age groups, who generally have a shorter duration of infection at the time of treatment, show earlier serological responses and a higher probability of seroconversion after treatment. In contrast, adults with chronic infection may require many years to reach negative seroconversion, even after parasitological clearance. Therefore, clearance of parasitemia and seroreduction of *T. cruzi* antibodies appear to be valuable predictors of future negative seroconversion. This proposed marker of treatment response (negative parasitemia and seroreduction) was clearly observed in our study, even when it was evaluated 1 year after the end of treatment. It is also noteworthy that the only patient with persistent positive parasitemia (therapeutic failure) showed no seroreduction.

In 2020, the United States’ Food and Drug Administration (FDA) accepted a decay of 20% in *T. cruzi* antibody titers combined with persistently negative parasitological tests as an efficacy biomarker in the registration of nifurtimox for the treatment of Chagas disease (30). That threshold for seroreduction was based on the results of a randomized clinical trial conducted in pediatric patients with Chagas disease, in which a 21% reduction in optical densities measured by conventional ELISA was observed in patients aged 6–12 years treated with 60-day benznidazole at 12-month follow-up (27).

The advent of PCR in the 1990s revolutionized the evaluation of treatment responses in Chagas disease. PCR is a sensitive and highly specific method for detecting parasitemia in the acute phase of infection, particularly in newborns, infants, and immunocompromised patients. In patients with chronic indeterminate Chagas disease, PCR has lower sensitivity as an indicator of active infection, and up to 60% of patients may have negative PCR at diagnosis. A positive PCR result during follow-up indicates treatment failure, making PCR valuable for confirming true-positive cases (31). However, because parasitemia in the chronic phase is low and likely intermittent (32), a negative PCR result does not necessarily confirm parasite clearance. PCR was introduced during the study period and therefore was not uniformly available across the entire cohort. Nevertheless, its introduction did not change the interpretation of treatment outcomes, because parasitological response was interpreted together with serological trends, and negative PCR results alone were not considered definitive evidence of cure. In our study, most treated patients achieved parasitological clearance during therapy, suggesting the potential for shorter treatment regimens. Recent studies have also explored alternative regimens with shorter durations and lower doses to improve treatment tolerability and adherence (27, 33, 34). Of note, in line with updated national guidelines (35), adult patients in this study received a 30-day treatment regimen with positive outcomes.

There is still significant debate about the treatment response criteria within the scientific and clinical communities that treat patients with Chagas disease. Earlier biomarkers of treatment response are needed. Some have been proposed in recent years, such as using non-conventional serological techniques. In particular, the MultiCruzi test (36) and specific biomarkers like anti-α-Gal lytic (37, 38) antibodies known as anti-F2/3 antibodies (29, 39) are being evaluated in adults and children. Children are the gold standard population for the validation of serological tests, as they have a high seroconversion rate. These new biomarkers should allow us to evaluate the treatment response for both children and adults more reliably and in a shorter time scale.

We observed a low incidence of cardiological lesions related to Chagas disease in treated patients (40), contributing evidence that antiparasitic treatment could decrease the risk of Chagas disease progression (16, 17). In a previous study from our group, 24-hour Holter monitoring and speckle-tracking two-dimensional echocardiography were performed in 234 patients from this cohort, and changes compatible with Chagas cardiomyopathy were identified in only four patients (40).

This study has several limitations. First, because it was a retrospective analysis of real-world clinical practice, there are limits to the conclusions that can be drawn. Although patients were managed and followed according to a standardized institutional protocol, visit schedules and diagnostic methods may not have been entirely uniform over the long study period. Accordingly, causal associations cannot be firmly established. Nevertheless, the extended duration of follow-up and the availability of repeated diagnostic assessments across multiple visits help mitigate some of these limitations and support the robustness of the observed trends. Second, although serological follow-up was consistently performed using ELISA throughout the study period, changes in commercial kits or manufacturer antigen batches may have occurred over time. However, these changes are unlikely to have substantially affected the overall interpretation of the results, because treatment response was assessed on the basis of longitudinal serological trends rather than isolated measurements. Third, PCR was introduced during the study period, limiting its availability for earlier patients. However, PCR has limited sensitivity in chronic Chagas disease. It is mainly used to detect treatment failure and is not considered a stand-alone marker of cure; therefore, its introduction did not affect the interpretation of parasitological responses. Fourth, our cohort reflects the clinical population of a national reference center for congenital and pediatric Chagas disease in an urban area, Buenos Aires City, without vector transmission. Accordingly, pediatric patients, particularly congenitally infected children, are overrepresented, and most adult patients were women, mainly mothers of index pediatric cases. This limits the external validity of the findings for the general adult population. Nevertheless, the results are highly relevant to settings without vector transmission, where congenital infection is the main route of transmission, and to children in endemic areas, where *T. cruzi* infection is also commonly acquired early in life. Finally, the follow-up analysis was based mainly on parasitological and serological biomarkers rather than on clinical outcomes, such as electrocardiographic changes, cardiac events, or death. Thus, although parasitological clearance and seroreduction are currently the main standards used to evaluate treatment efficacy, further studies with extended follow-up are needed to determine the extent to which these biomarkers correlate with the prevention of end-organ complications, particularly in adults. Of note, such studies are methodologically challenging and costly, posing a substantial barrier in the context of one of the most neglected diseases, for which research funding remains extremely limited.

In conclusion, our study contributes substantial data to the understanding of the effects and outcomes of benznidazole treatment in children and adults with Chagas disease. Benznidazole induced parasitic clearance in both age groups, reinforcing that *T. cruzi* infection is curable if treatment is initiated soon after infection.

## MATERIALS AND METHODS

### Study design and population

This is a retrospective age-stratified cohort study that assesses the long-term effectiveness of orally administered benznidazole in individuals with Chagas disease. Patients were treated and followed up at the Parasitology and Chagas Service, Hospital de Niños Ricardo Gutiérrez, Buenos Aires, Argentina, from January 1980 to July 2019. The Parasitology and Chagas Service at Hospital de Niños Ricardo Gutiérrez serves as a national reference center in a tertiary care–level pediatric hospital in an urban setting where there is no vector transmission. The service mainly handles referrals of newborns whose mothers were diagnosed with Chagas disease through the national pregnancy screening program. Often, these newborns act as “index cases,” enabling full family screening, and once infected relatives are identified, they receive treatment. As a result, most adult patients at our facility are the mothers of these newborns. The study protocol was approved by the Ethics Committee of Hospital de Niños Ricardo Gutiérrez, Buenos Aires, Argentina. Given the retrospective nature of the study, the requirement for informed consent was waived by the ethics committee. All procedures were conducted in accordance with applicable institutional and national ethical standards and with the principles of the Declaration of Helsinki.

### Chagas disease diagnostic criteria

According to national and international guidelines, the diagnosis of Chagas disease was stratified by age. In infants younger than 8 months, direct observation of *T. cruzi* by a parasitological concentration method (MH testor XD) or a positive PCR result must be evidenced. For all remaining patients older than 8 months, two reactive serological tests need to be positive: these are an enzyme-linked immunosorbent assay (ELISA), an indirect hemagglutination (IHA), or direct agglutination (DA).

### Inclusion criteria

Children having at least 55 days of treatment or adults with at least 28 days of treatment with benznidazole.

### Chagas disease classification

According to standard disease classification, newborns are considered to have acute Chagas disease due to congenital transmission, while adult patients are typically classified as having chronic infection. This distinction allows for an age-based comparison that also reflects different stages of disease progression.

### Patient stratification

Patients were stratified according to the following age groups: 0–7 months, 8 months–1 year, 2–6 years, 7–11 years, 12–17 years, and adults (over 18 years) based on previous studies showing substantial changes in treatment response within these groups (19, 29).

### Treatment

The benznidazole treatment (100 mg, Radanil Roche or Abarax ELEA or 12.5 mg, ELEA, Argentina) was prescribed in doses of 5–8 mg per kg per day divided into two daily doses given for 60 days, according to national guidelines at the time of diagnosis (treatment length was reduced from 60 to 30 days at later dates in adults). An untreated pediatric control group was not included since it is considered unethical to withhold Chagas disease treatment from children. Enrollment of children started in March 1980, and enrollment of adults began in July 2004. Infant benznidazole doses were provided as fractionated tablets prepared by a pharmacist and administered with water or mother’s milk. Medication was provided to patients or their guardians in monthly batches, and adherence was assessed by counting the remaining tablets at each visit. Treatment was considered complete when patients took the medication for at least 55 days for children and 28 days for adults and ≥17-year-old teenagers. Safety/tolerability of benznidazole and adverse drug reaction (ADRs) profiles were evaluated and described in a previous study (22).

### Primary outcome

The primary outcome of the study was treatment effectiveness, defined as a sustained therapeutic response to benznidazole, assessed through three outcomes: (i) sustained parasitological clearance, defined as persistent negative detection of *T. cruzi* by MH, XD, or PCR at all post-treatment assessments; (ii) seroconversion, defined as complete negativization of all performed serological tests; and (iii) seroreduction, defined as a ≥20% reduction in *T. cruzi* antibody titers from baseline, confirmed in at least two consecutive follow-up assessments. Treatment failure was defined as *T. cruzi* detection by any parasitological method (MH, XD, or PCR) at any time after treatment completion.

### Follow-up

Baseline information was obtained at the beginning of the treatment. Visits were carried out at 7, 30, or 60 days and the end of treatment, every 3 months during the first-year post-treatment, and every 6–12 months thereafter.

### Serology

At each visit, *T. cruzi* serology was carried out by conventional *T. cruzi* ELISA, DA, or IHA. Serological results by ELISA were expressed as the ratio between the optical density value recorded for each serum sample and the cut-off value of the assay (*R* = OD-sample/OD-cut-off). IHA and DA results were expressed on an inverse log_2_ scale. Depending on their availability, ELISA tests were performed from 1995 onwards for diagnosis and post-treatment follow-up.

*Seroconversion* was defined when all performed serological tests became negative during follow-up.

*Seroreduction* was defined as a decline of at least 20% in *T. cruzi* antibody titers from baseline. This was measured by ELISA and expressed as a ratio (OD sample/OD cut-off) from baseline. For IHA and DA (semi-quantitative tests), we used a 20% decay in the log_2_ of the number of dilutions.

### Parasitemia

For infants younger than 8 months, MH or XD was initially used for diagnosis and repeated weekly until negative results were obtained. From 1999 onwards, PCR samples consisted of peripheral blood mixed with an equal volume of guanidine hydrochloride 6M EDTA 0.2M buffer, pH 8.00. DNA was obtained from 300 μL of guanidine-EDTA-blood samples using the High Pure PCR Template Preparation kit (Roche Diagnostics Corp., Indianapolis, IN). A duplex real-time PCR (qPCR) assay targeted to *T. cruzi* satellite DNA and human RNase P gene was performed with 5 μL of DNA extract in a final volume of 20 μL, using the FastStart Universal Probe Master Mix (Roche Diagnostics GmbH Corp., Mannheim, Germany) and the TaqMan RNase P Control Reagents Kit (Applied Biosystems, Foster City, CA), as previously described (Ramirez & Moreira, 2019) (31, 41–43). (ii) For subjects older than 8 months, parasitological tests were not systematically performed at baseline, as diagnosis was based on serological tests. (iii) For all subjects, PCR was conducted intermittently throughout follow-up.

### Clinical evaluation

A complete clinical check-up was performed at diagnosis and every subsequent visit.

### Data collection

Data were collected from medical records of treated patients and entered into an Access clinical database (ACD) designed for this study. All individual data sets were anonymized.

### Statistical analysis

The rate and time of seroconversion and seroreduction were estimated using Kaplan–Meier survival analysis. Comparison among age groups was evaluated using the log-rank test and Cox regression. Statistical analysis was carried out using R software.
